# Exploring the Utility and Personal Relevance of Co-Produced Multiplicity Resources with Young People

**DOI:** 10.1007/s40653-021-00377-7

**Published:** 2021-07-27

**Authors:** Sarah Parry, Zarah Eve, Gemma Myers

**Affiliations:** Department of Psychology, Manchester Metropolitan University, Brooks Building, Manchester, M15 6GX UK

**Keywords:** Dissociation, Multiplicity, Dissociative identity disorder, Narrative

## Abstract

Multiplicity, the experience of more than one self in the body, is an under-researched area of young people’s mental health. The aim of this study was to explore the perspectives of experts-by-experience within a community sample regarding two specific resources: a co-produced self-help guide about multiplicity for adolescents, and a set of guidelines for supporting someone who identifies as ‘multiple’. 34 participants (M*age*= 22.06, 2.26 SD; 15F, 1M, 18NBG) completed an online survey consisting of open-ended and Likert scale questions to assess the language, utility, transferability and therapeutic impact of the materials. Descriptive statistics and a Foucauldian-informed Narrative Analysis were employed to analyse responses, producing a summary of utility and two narrative chapters. The emergent chapters, ‘Breaking the Stigma’ and ‘Recognising the Many’, highlight the need for greater understanding and awareness of multiplicity, with psychoeducation materials viewed as helpful. Inclusive language can reduce stigma and normalise multiplicity as a response to trauma. With greater understanding, practitioners and researchers can collaborate with young people through trauma wise care, providing multiplicity sensitive language and support. Overall, the term ‘parts’ was viewed as problematic by the participants as it could imply the plural system is not coexisting as a whole. Additionally, opinions varied as to how much diagnostic language could and should be used to describe multiplicity; linguistically and conceptually. Importantly, compassion was seen as particularly essential for younger selves within the system; older in their years and presence, but often more vulnerable within the societies in which the system resides.

Multiplicity has been defined as the experience of having two or more separate selves within one body (Spanos, 1994), with the body’s behaviour being controlled by one-self at any one time (Ribáry et al., 2017). Those who experience multiplicity often refer to themselves as multiples or systems (a system of separate selves; Ribáry et al., 2017). The separate selves within the system, otherwise known as ‘alters’, ‘parts’ or 'headmates’, usually have differing ages, genders, feelings, thoughts and memories (Dietrich, 2012). Henceforth, we shall refer to people with DID or multiplicity as ‘systems’ to recognise a more inclusive approach to language for people identifying as multiple.

Multiplicity can be placed on a continuum alongside dissociation and dissociative identity disorder (DID; O'Connor, 2016). While there may be some defined distinctions, phenomenological understandings of dissociation are often broad and lack clarity around individual experience (Naso, 2007; Spiegel et al., 2011; Parry et al., 2017). Definitional imprecision of basic components of multiplicity may destabilize the research around it and reduce the effectiveness of proposed pathways for care and support. Often, research discusses dissociation occurring as a result of childhood trauma, in which one multiple is the protector or means of escape within the child’s system of coping (Diseth, 2005; Read et al., 2014; Read et al., 2001).

In terms of differentiating between the terms DID and multiplicity, DID is associated with high levels of distress and reduced functioning within most diagnostic conceptualisations (American Psychiatric Association (APA), 2013). However, many people with multiplicity function well in terms of consciousness, memory, identity and perception of the environment (Ribáry et al., 2017), and appreciate the value of their multiple selves as a coping response to adversity and relational traumas (e.g. Parry et al., 2018a, b). The absence of distress experienced by systems identifying as multiple may suggest that DID and multiplicity vary in experience, and the dominance of DID in research highlights a fundamental limitation in the understanding of multiplicity (Okano, In Press; Trifu, 2019). On the other hand, an assumption of a lack of distress and impairment in functioning could point to a further lack of understanding in the experience of multiplicity (Hacohen et al., 2019; Sagan, 2019), particularly in relation to distress associated with stigma. Overall, the paucity of research surrounding multiplicity with people with lived experience, especially during adolescence and emerging adulthood, could alienate the multiplicity community, leading to important gaps in the scientific and humanitarian understanding of how systems of people can coexist together.

Due to a lack of research and clinical guidance, multiplicity can often be misdiagnosed and incorrectly treated (Wang et al., 2002). It is common for those with multiplicity to be wrongly diagnosed with a psychotic disorder (Spiegel et al., 2011). Individuals with DID can spend 5-12 years misdiagnosed with other conditions, often due to the lack of acceptance and awareness of dissociation from some professional groups (International Society for the Study of Trauma & Dissociation (ISSTD), 2011). This situation is particularly likely to affect young people as they are unlikely to have suitable access to care and support until later in adulthood. Further, many who experience multiplicity can function well in day-to-day life, so the assumption of pathology is questionable. If a young person’s presentation of multiplicity is pathologized and the young person is not supported to understand and formulate their own experience, they will have even less agency within the health care system. The prevalence of misdiagnoses and lack of accessible meaningful diagnoses suggests better methods are needed to recognise, validate, understand and facilitate useful therapeutic exchanges that empower young people who are likely to have trauma histories.

Parry et al. (2018a, b; Fig. 1) developed eight guidelines for compassionate communication with systems based on the accounts of five women with DID, who had experienced varied therapeutic relationships with healthcare staff on hospital wards. Since publication, this paper has been a ‘top ten’ most regularly downloaded mental health paper as reported by Elsevier. Based on citation metrics, the guidelines are already in the top 24% of the most cited work worldwide. These guidelines have been used by a number of National Health Service (NHS) inpatient services as no other specific guidelines for healthcare professions supporting multiple systems are available. The guidelines are promoted by a number of specialist trauma clinics and feedback from experts-by-experience has been positive due to the practical nature of the guidelines and their availability online. However, it is not currently known how useful these relational guidelines may be perceived to be by young people who self-identify as multiple. Young people identifying as multiple are largely absent from the trauma and dissociation literature, in part due to delays in diagnosis, treatment and limited opportunities to take part in research.

Therefore, the current study aimed to explore the utility and transferability of these guidelines with a younger demographic from a community sample, alongside a psychoeducation self-help guide about multiplicity, co-designed with young people at Voice Collective^1^. Voice Collective is a charitable peer-led group supporting young people with voices, visions, multiplicity and other unusual sensory experiences. The psychoeducation self-help guide was designed as both an informative resource for young people, as well as a communicative document they could use to help other people understand their multiple experiences.

Understanding is essential for empathy, validation and support. Both the resources have the potential to improve awareness, reduce stigma, and support better care for young people with multiple systems. Consequently, their suitability and relevance need to be sufficiently representative of young people’s experiences of multiplicity to be beneficial. Therefore, young people who identified as multiple were invited to provide feedback on the utility, language, transferability and potential therapeutic impact of these resources to develop them further. It was also hoped this process would inform research and practice with young people with emerging multiplicity or DID in terms of highlighting fundamental principles and key issues of importance.

## Materials and Methods

### Design

In addition to demographic and contextual questions, a qualitative design was used, allowing an in-depth exploratory enquiry into the psychoeducation self-help guide and guidelines (see Parry et al., 2018a, b). Qualitative methods were deemed appropriate as they are commonly used to elicit responses from the viewpoint of the participant about meaning, experience and opinions (Hammarberg et al., 2016); a key focus for the current study. An online survey was employed to promote convenience and inclusivity for participants (Parry et al., 2018a, b). Prior to the development of the survey questions, discussions were held with experts-by-experience to formulate key questions. The final survey underwent two reviews by transprofessional experts to refine the tone and structure.

### Procedure and Ethics

Following a full critical review of all participant facing documents by Voice Collective, the study was approved by an academic Research Ethics Committee. The first author shared the link to the Qualtrics survey with a brief summary of the research programme on social media. Voice Collective and designated social media groups for young people with multiplicity shared the survey link to ensure it reached our target popultaion. Participants could then self-refer to the Qualtrics site for further information and the survey. Prior to the start of each questionnaire, the participant read or listened to information about the study and completed a consent form. Survey questions were not mandatory and participants were advised some may be more relevant to them than others. Participants could also complete the questions at various time points prior to submission, resulting in a 46.7% completion rate of all questions, with all participants completing a self-selection of questions asked. To further promote inclusion with this under-represented group, participants could self-refer to the study if they experienced multiplicity, rather than being excluded if they did not have a dissociative diagnosis. If participants could only be included if they had a dissociative diagnosis, this would have been a major barrier as young people rarely receive timely support or diagnostic-based treatment, as discussed in the introduction.

Within the survey, participants were asked open-ended questions about the transferability (for example, to clinical staff), language, utility and the therapeutic impact of both the self-help guide and the guidelines for health staff. Specifically in relation to the guidelines for health staff, Likert scale questions were presented regarding the helpfulness and usefulness of each guideline to ascertain their relevance for young people. Finally, young people who completed the survey were asked about their experience of taking part in the study and if they had any final comments or recommendations.

A demographics questionnaire was included, collating age(s), gender(s), location and their chosen pseudonym, preserving confidentiality and anonymity (Table 1). At the end of the survey, participants were directed to a debrief page, with links to support and information included in case of distress due to the sensitivity of the study. Direct contact details of the researchers and Voice Collective were provided for additional support.

**Table 1:** Demographic characteristics of participants

	*n*	%
Age range		
16-19 20-22	6 14	17.1 40
23-25	15	42.9
Gender^a^		
Male	1	2.9
Female	13	37.1
Non-binary/Genderfluid	14	40
Transgender	4	11.4
Queer	2	5.7
Ethnicity^b^		
White	23	65.7
Hispanic	1	2.9
Maori	1	2.9
Chinese	1	2.9
African-American	1	2.9
Asian	1	2.9
Mixed Ethnicity	5	14.2

^a^1 non-response

^b^2 non responses

**Table 2:** Means and Standard Deviations for guideline usefulness scores

**Guidelines from **Parry et al. (2018a, b**)**	*n*		**Usefulness Score**			**% usefulness**
			M		SD	
G1: Younger parts may require additional acknowledgment, nurture and support	33	4.33		0.65		42.42% extremely 48.48% very 9.09% somewhat
G2: Avoiding a singular perspective on the self as a construct may be helpful, for example, enquiring “how is everyone?” may be preferable to “how are you?”	34	4.29		0.84		52.94% extremely 23.53% very 23.53% somewhat
G3: Younger parts may have particular difficulties expressing themselves verbally. Therefore, alternative methods of communication should be agreed with the main persona (e.g. toys, music or drawing)	32	4.25		0.92		53.13% extremely 21.88% very 21.88% somewhat 3.13% not very
G4: Different alters may require an introduction to staff known by the main persona, and vice versa	33	4.24		0.79		45.45% extremely 33.33% very 21.21% somewhat
G5: Compassionate acceptance and support for people with younger parts was identified as essential for the wellbeing of the whole person	33	4.39		0.83		57.58% extremely 27.27% very 12.12% somewhat
G6: Demonstrate authentic interest in the person’s wellbeing through asking questions and becoming educated around their individual condition. All participants highlighted what a difference staff training around complex trauma and dissociation would have made for their treatment, wellbeing and recovery.	33	4.70		0.53		72.73% extremely 24.24% very 3.03% somewhat
G7: Common ground was seen as being very important in order to develop relationships	34	3.85		0.89		26.47% extremely 38.24% very 29.41% somewhat 5.88% not very
G8: Participants often had difficulty recognising, remembering and locating their named nurse. Therefore, people with DID should be provided with an information card about their key staff including a photograph, name and perhaps some appropriately brief information about hobbies or interests	33	4.21		0.89		48.48% extremely 27.27% very 21.21% somewhat 3.03% not very

1=not at all; 2=not very; 3=somewhat; 4=very; 5=extremely useful

### Participants

The individual responses of 34 young people formed the data set of this study (M*age* = 22.06 years, *SD =* 2.26). To our knowledge, this is the largest qualitative study with young people with multiplicity to date. Inclusion criteria for those who took part was to be between 16 and 25 years old (adolescence to emerging adulthood) and to have direct experience of multiplicity. Due to the varied ages and genders of the participants’ individual multiple systems, most participants did not identify as a single gender overall. Participant quotes are included verbatim in the analysis to preserve their integrity. Due to existing disparities surrounding the understanding of multiplicity, the study did not exclude individuals based on the aetiology of their multiplicity and did not request information concerning a potential DID diagnosis. As a consequence of a lack of guidelines currently available, the aim was to engage with young people’s systems at all sections of the multiplicity spectrum. Commonality and volume in the returns within a two-week period suggests good credibility and dependability in the responses.

Additionally, it was evident that many participants enjoyed their participation in the research project. For example, *Emco (22)* stated, “This generally seems like a really amazing project, and the approach you’re taking towards multiplicity is a really positive change for psychological research”. This notion was supported by *Kris (21)* who said, “I really liked the leaflet and I'm excited to see some academic recognition of multiplicity that is understanding”. *Lia (19)* mentioned that they: “want there to be more resources than what I had available to me”, suggesting this is a good starting point that was valued by the plurality community.

### Analytic Approach

Foucauldian-informed Narrative Analysis (FNA) aims to examine how power intervenes in people’s thoughts and assumptions (Tamboukou, 2013). FNA recognises the top-down influences of social processes at a macro-level in people’s lives (Sam, 2019), such as stigma surrounding marginalised groups, whilst aiming to understand the diversities of meaning evident within individuals’ narratives (Burr, 2015). Importantly, FNA aims to illuminate the impact that individuals’ stories have on shaping meaning and perspectives on a given topic (Crossley, 2007; Murray, 2015; Tamboukou, 2013). As previously mentioned, not only is multiplicity an under-researched area, but so is the target age group. Therefore, the creation of the shared narrative in this study from young people presents a novel collective voice not previously heard. To the best of our knowledge, this is the first study to explore youth-focused multiplicity resources directly with adolescents who self-identity as multiple. The analyses upon the data relating to the guidelines and psychoeducation self-help guide were conducted together to explore the relevance, language use and utility of the two documents for young people experiencing multiplicity.

FNA brings focus to what is and is not said by participants and why it is said, to strengthen the researcher’s understanding, recognising the biomedical and socio-cultural influences around DID and multiplicity we are all exposed to. A critical constructivist approach was employed throughout the study, recognising a lack of clarity in the scientific conceptualisation of multiplicity and facilitating a constructively critical space for pre-existing assumptions and traditional ways of thinking. While other qualitative methodologies can also provide this, Willig (2013) suggests that IPA does not pay appropriate acknowledgement to the crucial role that language plays. An additional narrative hermeneutic in this study was that young people were providing perspectives and feedback on a document co-produced with other young people and guidelines developed by adults, although all shared experiences of multiplicity. Further, there were a range of age ranges within each participant’s multiple system, which adds further complexity to overarching narrative perceptions of the materials.

Foucault did not detail a precise method for approaching such research; instead the methods employed are highly dependent on the topic. As such, the current study utilised a six-step FNA framework specifically developed for qualitative research with young people who hear voices to recognise the multi-layered narratives within society, care systems and internalised by individuals surrounding stigmatised experiences such as multiplicity (Parry & Varese, 2020, S2). The process involved: (1) conceptualising origins at the individual level for the two documents; (2) orientation in relationships and construction, again within each document; (3) language and power in relationships within and across the data relating to the two documents; (4) features of individuality and commonality across the dataset; (5) constructing a resolution and finally (6) phenomenological and emancipatory narratives from across the whole dataset, interpreted through the analytic steps.

## Analysis and Discussion

### Descriptive Summary

Descriptive statistics were explored in relation to the Likert Scale data evaluating the guidelines for health staff (Tables 2 and 3). In terms of the previously published guidelines in particular, ‘demonstrate an authentic interest’ (G6) was ranked as the most beneficial guideline. *‘?’ (23)* said, “I particularly like the encouragement to ask questions and to not make assumptions”. A large body of research has identified the benefit that a positive therapeutic alliance has on patient reported outcome measures within a range of conditions including psychosis (Browne et al., 2019), eating disorders (Graves et al., 2017; Rienecke et al., 2016) and depression (Kushner et al., 2016; Labouliere et al., 2017). Within multiplicity, it is important for experts-by-experience to identify a trauma-informed therapist, particularly if the person’s plurality is traumagenic (McCartney et al., 2019; Parry et al., 2017). *Luca (24)* said *their* guidance to other systems would be to “try to find a trusted person you can talk to this about, preferably a trauma-informed therapist. Someone who can give you proper time”.

Guidance focused on younger headmates was also viewed as useful, particularly the need to treat younger headmates with compassion (G5). *Outlaws (25)* said, “We like the focus on different types of system mates, e.g. younger ones, and their individual needs”. This was supported by *Luca (24)* who explained “Sometimes younger parts are technically the older parts of a system [existed for longer], this can mean that they have important knowledge, skills, or roles within the system”. Therefore, support networks need to work with the various headmates to elicit information in a supportive manner and recognise their unique and important contributions, similar to family therapy sessions utilising different skills and tools on separate members (Gelin et al., 2018; Liederman, 2020). *The Network (25)* added, “Some younger parts in general are stuck in a traumatic time and need additional support to understand that they aren't in a bad place anymore”.

Facilitating common ground (G7) was identified as the least helpful guideline by respondents. This appeared to be due to the misinterpretation of the language used and miscommunication of the underlying meaning. *Salts (25)* said, “We don't really understand what the … guideline means. Common ground for what? Seen as important by whom? Develop relationships with whom?”. This was further supported by *Emco (22)* who said “The common ground point doesn't really make much sense to me”. This perhaps highlights one of the limitations of brief written guidelines for practitioners without further context. Within future guidelines, clarification and practical examples could facilitate clarity for those with plurality and practitioners.

### Foucauldian-informed Narrative Analysis

The analysis led to two distinct analytic layers that provide novel nuanced insights in to the language, utility, therapeutic impact and transferability, indicating the beneficial effect of the resources as well as the need for further improvements. Upon initially reviewing the data set at an individual level, within individuals and then across individuals, eight emerging narrative layers were identified. These were: 1. The resources included a clear explanation of multiplicity, which utilised unambiguous inclusive language, which allowed respondents to feel understood; 2. The resources were a good starting point to be developed further; 3. The coping strategies presented were beneficial, and should be added to and further separated into sections; 4. Triggering language choices, in particular “parts”, which invalidated the target audience’s experiences; 5. Ongoing stigmatisation from the media, which presents multiplicity in a factually inaccurate, damaging way; 6. The non-pathologising approach was appreciated, with no hardcore diagnosis terminology, suggesting multiplicity does not always originate from trauma; 7. Overly simplified and needing more technical information, and a heavier focus on trauma and Other Specified Dissociate Disorders [OSDD]; 8. A need to separate the study of multiplicity from DID and OSDD.

These eight layers were then further analysed and synthesised to form the two phenomenological and emancipatory narratives. ‘*Breaking the Stigma’* emerged as a narrative layer primarily concerned with the benefits experienced from the resources that establish a normalizing language around multiplicity that a general community can appreciate. ‘*Recognising the Many*’ emerged as a second narrative layer concerned with the concept that there are many varying wants and needs across the population that are not sufficiently addressed within the leaflet or guidelines, highlighting the complexity of multiplicity.

### Breaking the Stigma – “It is not something that should be stigmatised” – Rubidah (23)

Multiple thematic narratives were identified that clustered around a narrative of normalising multiplicity. Respondents felt the resources had clear explanations of multiplicity, which utilised inclusive language. As such, the respondents felt they were helpful tools for both those with experiences of multiplicity and the people they interact with. Additionally, participants noted the aspect of the resources providing easily understood coping strategies that, when combined with the good explanation of multiplicity, was recognised as a beneficial starting point in helping others understand the experience. Finally, there was commonality across participants seeing the recognition of multiplicity in published material and this helping to reduce the stigma of multiplicity.

A recurrent narrative was that many felt that multiplicity was negatively viewed by society due to a lack of understanding and stigma in the public domain. *Heptagram (20)* said, “It isn’t inherently bad or evil or criminal”, and *Ruby (20)* stated “We aren’t murderous psychopaths that the media portray us to be”. This was brought up many times across participant narratives, demonstrating commonality and that in many systems’ experiences, multiplicity was often misunderstood and negatively viewed. *Kris (21)* explained “I really liked the leaflet and I’m excited to see some academic recognition of multiplicity that is understanding” and *Lia (19)* said “[the guidelines] would have changed my therapy experience for the better if they had been in place where I go”. Internalisation of stigma can result in depression and increased anxiety (Penn & Martin, 1998), highlighting the importance of removing stigma. As noted by Crabtree et al. (2010), increased social support can act as a buffer against stigma, resulting in increased self-esteem, while society’s understanding is modified. Utilising resources such as those examined in this study could facilitate enhanced understanding within the public domain. This, essentially, can work towards reducing the stigma around multiplicity and therefore allow individuals to feel understood, reducing avoidable distress and associated mental health difficulties.

Historically, there has been a stigma attached to various mental and physical health difficulties, such as depression, suicidality, and HIV (Barney et al., 2006; Duffy, 2005; Oexle et al., 2019). An increase in public understanding of these experiences has resulted in a focus on reducing the associated stigma (Thornicroft et al., 2016). This has been facilitated by positive stories being told within various media systems that incorporate such disorders (Niederkrotenthaler et al., 2014). As such, those with experiences of multiplicity hope for normalisation to occur within public perception. *The Network (25)* states they would like to see an “acknowledgement that multiplicity can be normal for some people, that it isn't inherently suffering”. An increase in knowledge, or ‘mental health literacy’ has been deemed a protective factor against negative stereotypes (Thornicroft et al., 2016). Thus, an increase in academic literature, and publicly disseminated information could aid in stigma reduction. Furthermore, normalisation of multiplicity would reduce forms of discrimination many in the plurality community experience. Recently, discrimination has been used to refer to emotional, cognitive and behavioural aspects of stigma (Campbell et al., 2013), such as the media and/or general public misinterpreting the experience of multiplicity. *Angela (22)* stated “Multiples are not crazy or incapable, our brain is just supporting us in the way it knows best how to do it”.

When considering the language of the leaflet, *Kris (21)* said “I liked a lot of the language that was used and I can tell the language choices were very carefully chosen and I appreciate that”. *Solar System (19)* supported this notion by saying “I felt included [...] the information was accurate and diverse”. A meta-analysis conducted by Griffiths et al. (2014) found that public stigma-reduction interventions such as educational resources reduced stigma associated with mental illness. Resources such as the leaflet can aid the reduction of stigma associated with multiplicity, while the guidelines could clearly and accessibly used by people more widely. Mindful awareness of language choices and how they can affect the reader is important as if there are seen to be inappropriate or ‘triggering’ language choices, it can deter readers and so the tool could become void. This is clearly demonstrated by *Outlaws (25)* who said, “we had to skim through much of the flyer” due to finding a particular language choice “triggering…as many individuals in our system have a long history of being dehumanized”. It can be said that the transferability, as well as the utility and therapeutic impact of the leaflet and guidelines, suffer if the language choices are not inclusive and carefully chosen. Upon consideration of the language used, changes are required to ensure inclusivity within the plurality community, in particular the phrase “parts” was seen as damaging to many respondents. *ACE (22)* explained “[it] can make the members of a system sound like ‘part of a whole’ and therefore not as important as other humans”. This notion was supported by *Amethyst (22)* who said “’Parts’ implies that one’s alters are not full people, which can feel invalidating”. *H. Chord (24)* hopes that future resources focus on the fact that “’parts’ might have their own self-identities outside of just ‘parts’”. However, as respondents appeared to view the language of the resources as generally positive and inclusive, the resources can clearly provide positive therapeutic impacts and help to reduce stigma, although the term ‘parts’ appears problematic.

It was suggested that the leaflet stood as a “good starting point” *(Sasha, 22)* in the area of multiplicity, and a “good way to introduce concepts of many different multiplicity experiences, away from an overload of jargon [...] that might make people feel confused or alienated” *(Sasha, 22).* This may suggest that previous explanations or support given around multiplicity has not been sufficient or beneficial and may have left people feeling isolated and stigmatised. It has been found that for those with a mental health disorder to develop a positive self-image, they need to feel accepted (Erdner et al., 2005) and if systems feel alienated due to clinical ‘jargon’, then this cannot be achieved. Due to the large discrepancies within multiplicity prevalence rates, utilising non-pathologising language can allow more individuals to feel understood by the resources (Loewenstein, 2018). *H. Chord (24)* said “I feel that this does a good job of covering the basics in an inclusive and non-alarming way”. This further develops the possible utility of the leaflet acting as a good starting point for individuals, whilst also making reference to the inclusive language used: “I think overall the leaflet does a good job of being an overview for people who are unfamiliar with multiplicity to get a grounding that they can later build upon and refine.” (*Team Spirit, 24).*

Collectively, the empowering themes of being understood, a positive and inclusive language and the creation of publicly accessible material that recognises, rather than alienates, multiplicity consistently emerged as an emancipatory motif of the narrative analysis and could be further developed to help reduce the public stigma associated with multiplicity.

### Recognising the Many – “The populations have quite different needs” – Luca (24)

The second analytic layer illustrates the intrinsic complexities in developing transferable, generalisable materials for people who experience multiple populations within themselves. Participants expressed their recognition of individual needs, whilst also discussing a need for common language and communicating throughout the system. This layer was characterised by the triggering language choices and varying needs, wants and opinions across the participants’ populations.

Highlighting important insights into how language should be considered carefully, participants stated that they found most of the language used in the psychoeducation leaflet and guidelines positive and inclusive, although many participants also felt that some specific language choices were triggering. For example, *ACE (22)* said “‘Parts’ is a language choice that can make the members of a system sound like ‘part of a whole’ and therefore not as important as other humans.”. When asked what would be wanted from future research, *Klaus (19)* said “I just want to see it actually validated”. As previously mentioned, being understood is important for wellbeing (Johansson & Eklund, 2003; Shattel et al., 2006) and can lead to validation and acceptance. Psychological acceptance is vital as a lack of it has been found to be associated with a wide array of mental health difficulties (Bond & Bunce, 2003), highlighting the need for these resources to be utilised in a helpful manner.

Regarding the leaflet specifically, *Outlaws (25)* said, “We find terminology such as ‘parts’ to be highly triggering…. There is no ‘whole person’ in our system, we are all ‘whole people’ and should be treated as such”. Such statements indicate some important disparities between how young people with multiplicity experience themselves and how the scientific community have perhaps mis-conceptualised the experience due to a lack of first-person accounts. This view is further recognised by other participants, with The *Network (25)* stating, “it contributes to people using a singular perspective towards multiples, which the guidelines also say to try to move away from”. In this context, the use of the word ‘parts’ could be seen as non-accepting, as the participants indicate, a part of something could be seen as *lesser than*. Therefore, this is perhaps not seen as inclusive language for young adults who are still at a developmental life stage of forming a full sense of themselves. Major and O'Brien (2005) suggest that stigma can threaten an individual’s personal and social identity, therefore creating involuntary stress that requires coping strategies to manage. As language plays a large role in the (de)stabilisation of understanding, much more direct engagement with experts-by-experience is needed to improve existing materials if they are to be effective for those experiencing plurality (Nijenhuis & Van Der Hart, 2011).

A further recurrent message within the participant narratives was that of the varying wants and needs across the population. This was made evident through conflicting input from respondents stating that aspects were both positive and triggering, with little agreement upon some areas of language use. For example, when asked what aspects worked well in the leaflet, *The Draco System (23)* said “No hardcore diagnosis terminology”. However, *Rabbit (22)* explained it was “a little bit condescending, could have had more psychological terminology” and *‘?’ (23)* said “It is too simplifying”. Therefore, while participants appreciated the lack of pathology, it still seems important to contextualise the experience in some scientific language, perhaps to add validation to and recognition of a condition that some clinicians still observe with some scepticism. However, with this study having established that different populations constructively responded to these materials, it is now increasingly possible to further develop suitable materials and build upon these unique and valuable insights.

The varying preferences and needs of the participants’ systems are further showcased by *‘?’ (23)* stating that “to readers who have no previous knowledge of multiplicity, this may read as though the experience isn’t distressing or that it ‘isn’t a big deal’ and may even feed into misconceptions and stigma surrounding DID/OSDD”. Consequently, while many participants found the resources beneficial, and even found them to challenge social stigmas, *‘?’ (23)* clearly demonstrates that not only does everyone have a different understanding of the experience of multiplicity but also have different opinions as to how it should be presented linguistically and conceptually. This further highlights the need for more individualised information around multiplicity and for generalised materials to represent the breadth of the experience and range of emotions associated.

In addition to feedback on language use and multiplicity-related distress, the aetiology of multiplicity was also discussed with varying perspectives across the participants. As aforementioned, some report multiplicity is traumagenic (Ellason & Ross, 1997), while others suggest that individuals are born with multiple capacity (Ribáry et al., 2017). This divergence was mirrored in the participant’s responses. For example, *Kris (21)* said, “I was excited when I found out that it doesn’t have to be related to trauma” and *The Network (25)* added, “I really like that there’s the mention that sometimes multiplicity starts from something bad, like intense childhood trauma, but it is not always like that”. Both participants support the idea that multiplicity isn’t solely traumagenic; that it can stem from a variety of circumstances, and they appreciate that the leaflet acknowledges this diversity. Conversely, when asked what their key message would be to help others understand multiplicity, *Rabbit (22)* stated, “It is always post-traumatic” and when speaking of the guidelines, *The Garden Groves (23)* said that the “emphasis on trauma-informed practice is good”. However, *‘?’ (23)* said that they “wouldn’t want to encourage or spread the belief that multiplicity is possible without trauma, certainly not in the way that this leaflet does”. This clearly indicates further development work with the multiplicity community is needed to explore whether such positions can co-exist within the term ‘multiplicity’, or perhaps whether the occurrence of trauma and multiplicity-related distress may need an alternative term. Due to the common agreement that DID is seen as too diagnostic amongst the young adults who took part in this study, it may be than an alternative suitable for adolescents and young adults may be required.

This point of contention is further discussed by *Luca (24)* who reflected, “I’m not sure if it’s a good idea to lump natural multiplicity together with DID/OSDD” and *Solar System (19)* said, “There is a dire need to study multiplicity outside of DID in general”. Here, a strong need is shown for more research and resources solely on multiplicity, separate from DID. Overall, participants recommend further research to address the many uncertainties, unknowns and questions left to explore: “Make sure to ask which accommodations a system needs” *(Solar System, 19).*

## Concluding Discussion

In summary, two narrative layers emerged regarding the utility, language, transferability and therapeutic impact of the guidelines and leaflet examined by the respondents. ‘Breaking the Stigma’ highlighted the importance of normalisation, inclusive language and positive understanding from support networks when aiming to reduce the stigma currently associated with multiplicity and dissociation. As there is currently a paucity in resources available, both those with plurality experiences and support networks lack adequate information and guidance, further limiting the normalisation of the experiences in the public domain. Consequently, respondents suggested the resources examined would be beneficial with minor alterations made to the language to ensure young people within the plurality community feel included and recognised. A synthesis of these transferable recommendations is provided in Table 3. These recommendations could guide researchers and practitioners in the development of future research, co-production, public health information and awareness campaigns.

**Table 3:** Respondents’ recommendations for changes regarding the resources

**Positives of the resources**	**Recommendations for changes to the resources**
The resources use open, non-pathologising language, which the plurality community commend.	The resources should remove triggering language such as ‘parts’, which are detrimental to the community.
The resources include some good advice that young people can benefit from.	Guideline six and eight need to be reworded to make them easier to understand.
Participants liked the explanation of different experiences, and that not everyone’s multiplicity is traumagenic.	The resources could make it clearer that headmates are separate and cannot control each other.
Participants liked that the resources focused on the individual needs of different headmates.	Links can be made between child headmates and headmates that are non-verbal or neurodivergent; not only child headmates may benefit from alternative methods of communication.
Participants felt the resources will help people in the plurality community feel understood, which hopefully will reduce the stigma attached.	The resources could make it clearer that not all systems have a ‘host’, and that sometimes the host is not the most knowledgeable headmate.
It is good to see collaboration with experts-by-experience to allow all resources to have those in the plurality community at the centre.	Future resources need to be specific for multiplicity and DID/OSDD instead of a combined resource. This will allow for all in the plurality community to feel supported and validated.

‘Recognising the Many’ demonstrated that while these resources were beneficial and important to the multiplicity community, there is still much research to be done in this area. Varying needs were identified across the participants, such as a desire for and against a traumagenic focus, a wish for more and less research on integration, an appreciation for simplicity and inclusivity as well as possibly being reductionist. There is much to be done regarding how to best offer a range of support to meet the varying and complex needs of these young people. Due to a lack of understanding of the condition, there is limited information and resources that accurately reflect emerging multiplicity for young people. This research goes some way to address these gaps. In essence, amongst our participants, the journey between multiple occupation and accepted coexistence within the system was ongoing, making the use of language and encouraging sociocultural understanding especially important. This could be due to the transformational and fluid nature of the self during adolescence, in addition to the development and consolidation of the multiple system. As seen in Table 3, the participants identified many positive aspects of the resources, which could also translate to wider practices and resources in this area.

While there remains a need for further clarity regarding the phenomenological and aetiological experiences of multiplicity and DID for young people, this study has developed some novel insights into how those with personal experiences perceive and often thrive with the occurrence of plurality. Additionally, some key guidelines have been identified that further understanding and language use. It is clear the plurality community have important insights for future research and the co-production of materials. Overall, the information from participants could inform tailored guidelines for therapeutic engagement and positive communications with young people who identify as multiple, presented in Fig. 2.

The online survey methods employed in this study facilitated the inclusion of young people who may not have been able to travel to take part in face-to-face interviews or focus groups held in a small number of locations, although the opportunity to dynamically engage with participants conversationally was missed. Further, despite the peer review of questions and helpful contribution from experts in the field around questions design, the participation rate of some questions was lower than others, perhaps indicating again the breadth and depth of these experiences means language choice can be difficult to achieve successfully for all. Future studies may benefit from the use of discussion, in person or through digital teleconferencing software, to allow for clarifications to be made and to offer the ability to request further information and elicit more detailed responses. The implementation of a combined online questionnaire and interview methodology would enable greater data saturation to occur.

## Recommendations for Trauma-Informed Care

Further research into the various experiences of young people within the plurality community is required. Due to the trauma histories shared by many young people with multiplicity, a sensitivity to language and validation is particularly important. Further, guidelines that promote safety, connections and coping are essential for trauma wise care and therapeutic relationships (Bath, 2015; Parry et al., 2017). These principles, alongside empowering co-production with young people, are important for developing practices that encourage young people’s agency and voice in the process. These are also key aspects of trauma-informed care. It has previously been documented that young people with high exposure to adversities may be less likely to engage in typical health services, although trauma-informed care could provide a more accessible option (Powers et al., 2015). “TIC involves validation and recognition of the effects of traumatic events, common coping strategies, and effective treatments” (Oral et al., 2016, p.330). Organisational and cultural changes are needed to underpin and support consistency for trauma-informed care. An adoption of trauma-specific assessments and interventions could expediate the treatment process for young people with emerging multiplicity and address the commonly reported trauma histories of these young people.

Additionally, having a condition such as multiplicity that is not generally validated and recognised in a community can be, in itself, traumatic. Therefore, even if trauma does not precede the development of multiplicity, trauma-informed care would still be appropriate for many young people who seek help for multiplicity. Consequently, a separation of multiplicity and DID within both research and language could help improve understanding and the delivery of care. This more nuanced understanding of multiplicity could work in tandem with producing tailored information for different populations. Considered community-based co-production to expand knowledge with experts-by-experience in this area could help to develop beneficial strategic alliances and reduce stigma surrounding multiplicity and DID, particularly for those at the fragile transitional developmental stage of adolescence and emerging adulthood.

**Fig. 1:**
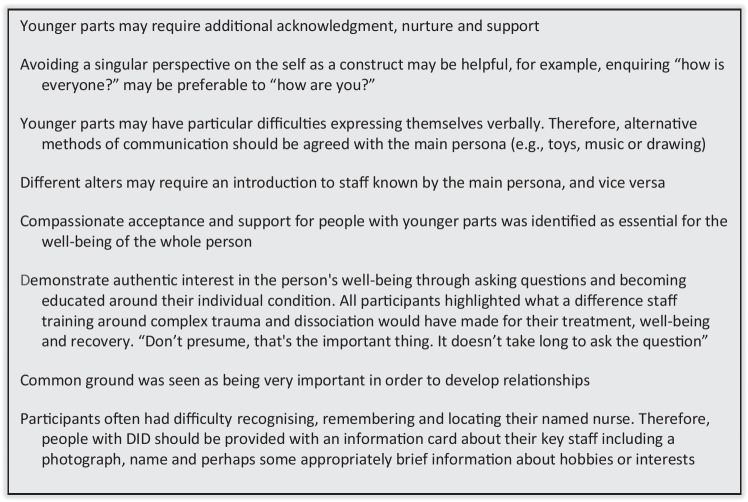
2018 Guidelines from https://doi.org/10.1016/j.ejtd.2017.08.002

**Fig. 2:**
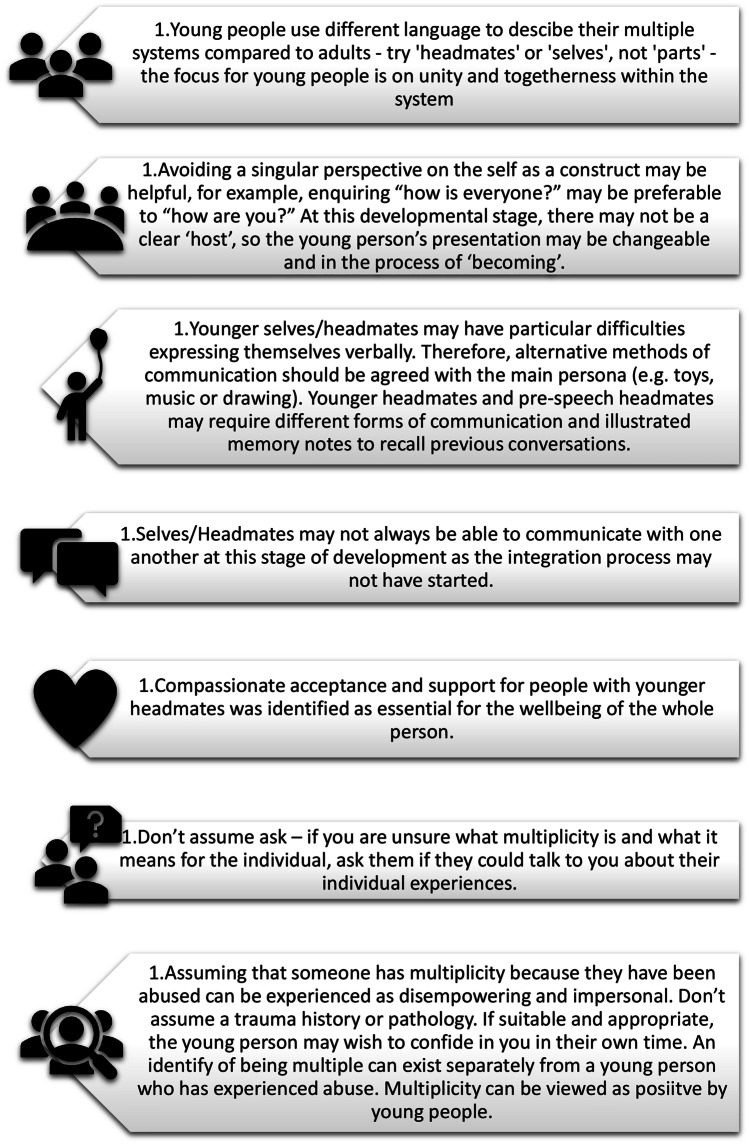
Guidelines for therapeutic engagement and positive communications with young people who identify as multiple

## Footnotes

1 Available at: https://www.mmu.ac.uk/media/mmuacuk/content/documents/hpsc/research/projects/young-voices-study/19189-THE-Awards-Multiplicity.pdf
